# Molecular epidemiology and viremia of porcine astrovirus in pigs from Guangxi province of China

**DOI:** 10.1186/s12917-019-2217-x

**Published:** 2019-12-27

**Authors:** Yifeng Qin, Qingli Fang, Xunjie Li, Fakai Li, Huan Liu, Zuzhang Wei, Kang Ouyang, Ying Chen, Weijian Huang

**Affiliations:** Laboratory of animal infectious disease and immunology, College of Animal Science and Technology, Guangxi University, No.100 Daxue Road, Nanning, 530004 People’s Republic of China

**Keywords:** Porcine astroviruses, Pigs, Genetic diversity, Viremia, Guangxi province

## Abstract

**Background:**

Porcine astroviruses (PAstVs) are common in pigs worldwide. There are five distinct lineages with each lineage representing a different ancestral origin. Recently, multiple reports have demonstrated the evidence of extra-intestinal infection of PAstVs, but little is known about viremia.

**Results:**

In this study, a total of 532 fecal samples and 120 serum samples from healthy pigs were collected and tested from 2013 to 2015 in Guangxi province, China; of these 300/532 (56.4%) and 7/120 (5.8%) of fecal samples tested positive for PAstVs, respectively. Our study revealed that there was wide genetic diversity and high prevalence of the virus in the pig population. All five of the known PAstVs genotypes (1–5) prevailed in the pig population of Guangxi province and were distributed in all age groups of pigs, from suckling piglets to sows, with PAstV2 (47.7%), PAstV1 (26.2%) and PAstV5 (21.5%) seen predominantly. Phylogenetic analysis of partial ORF1b and partial capsid sequences from fecal and serum samples revealed that they were divided into the five lineages. Among these genotypes, based on partial ORF2 genes sequencing 23 strains were grouped as PAstV1, including 6 serum-derived strains, and were regarded as the causative agents of viremia in pigs.

**Conclusions:**

Due to the information regarding the types of PAstV in blood is limit. This is the first report for the presence of PAstV1 in blood and PAstV3 in the feces of nursery pigs of China. This study provides a reference for understanding the prevalence and genetic evolution of PAstVs in pigs in Guangxi province, China. It also provides a new perspective for understanding of the extra-intestinal infection of PAstVs in pigs.

## Background

Astroviruses (AstVs) belonging to the family Astroviridae was first identified by electron microscopy (EM) in fecal samples from children suffering with diarrhea in 1975 [1]. To date, AstVs are divided into two genera: *Mamastrovirus* and *Avastrovirus*, which contain 19 and 3 species, respectively [2]. AstVs are single-stranded positive-sense RNA viruses, about 28-30 nm in diameter, whose genome contain three open reading frames (ORFs), namely ORF1a, ORF1b and ORF2. ORF1a and ORF1b code for non-structural proteins, a protease and a RNA-dependent RNA polymerase (RdRp), respectively [3]. ORF2 codes for a highly divergent capsid protein which is formed in response to the immune pressure from the host [3]. Moreover, the genome also includes a 5ˊ-untranslated region (UTR), a 3ˊ-UTR and a poly-A tail.

Generally, AstVs were considered to be enteroviruses, causing diarrhea in most of the sensitive mammalians. In particular, human astrovirues (HAstVs) have been recognized as the second most common cause of viral diarrhea in young children, the first being rotaviruses [3]. Porcine astroviruses (PAstVs) belong to the genus *Mamastrovirus* and were identified for the first time in 1980 by electron microscopy [4]. Based on the phylogenetic analysis of the full length ORF2 capsid protein, PAstVs are divided into five genotypes (PAstV1-PAstV5) circulating in the worldwide [5–9]. In 2010, the International Committee on Taxonomy of Viruses (ICTV) classified PAstV1 as *Mamastrovirus* 3 [10]. However, the other four genotypes of PAstVs have not been confirmed to exist in which species of *Mamastrovirus* [10]. Astroviruses are detected relatively more often in diarrheic pigs as opposed to healthy ones [11, 12], but there is lack of more evidence to clarify the direct or indirect association of PAstVs with diarrhea [13]. Besides, there was more evidence of extra-intestinal infection of AstVs, it is worth noting that AstVs are detected in the central nervous system (CNS) presenting with encephalitis in various cases involving humans [14–16], mink [17], cattle [18–20], pigs [21, 22] and sheep [23]. Additionally, PAstVs were also detected in serum [24, 25] and in the respiratory and circulatory systems of animals [24, 26].

The prevalence of PAstVs in most pig herds of China is still poorly documented, especially as little is known about viremia. Therefore, the main objective of our study is to provide more data on the prevalence of different types of PAstVs in Guangxi province and bring new knowledge with respect to infection in extra-intestinal system.

## Results

### Detection of PAstVs in pig serum and fecal samples

In this study, all the samples were tested for the existence of PAstVs by the nested RT-PCR method. The positive rates of PAstVs in feces samples reached 56.4% (300/532), in pigs of different developmental stages which showed divergent prevalence, including 71.0% (103/145) in nursery pigs, 58.2% (82/141) in suckling pigs, 53.3% (65/122) in finisher pigs and 40.3% (50/124) in sows (Table 1). Interestingly, there were 7 PAstVs-positive samples tested in 120 serum samples, including one (belonging to PAstV1) on each from suckling pig and nursery pig, respectively and five from sows belonged to PAstV1(4/5) and PAstV2(1/5). The PAstVs-positive prevalence in serum samples reached to 10% in farm A and 1.7% in farm B (Table 1).

**Table 1:** Results for detection of PAstVs in fecal and serum samples

Age groups	No. samples	Positive rate (%) of fecal samples	No.clones^a^/PS	Positive rate (%) of the samples sequenced (2013–2015)					Positive rate (%) of serum samples (2015)	
				PAstV1	PAstV2	PAstV3	PAstV4	PAstV5	Farm A	Farm B
Suckling pigs	141	58.2 (82/141)	19/82	31.6(6/19)	42.1(8/19)	0 (0/19)	10.5(2/19)	15.8 (3/19)	0 (0/20)	5.0 (1/20)
Nursery pigs	145	71.0 (103/145)	19/103	26.3(5/19)	47.4(9/19)	5.3(1/19)	0(0/19)	21.1 (4/19)	10.0 (1/10)	0 (0/10)
Finisher pigs	122	53.3 (65/122)	10/65	20.0(2/10)	60.0(6/10)	0 (0/10)	0 (0/10)	20.0 (2/10)	0 (0/10)	0 (0/10)
Sows	124	40.3 (50/124)	17/50	23.5(4/17)	47.1(8/17)	0(0/17)	0 (0/17)	29.4 (5/17)	25.0 (5/20)	0 (0/20)
Total	532	56.4 (300/532)	72/300	26.2(17/65)	47.7(31/65)	1.5(1/65)	3.1(2/65)	21.5 (14/65)	10.0 (6/60)	1.7 (1/60)

^a^ refers to the sequence of phylogenetic analysis in Fig. 1; PS indicates positive samples

### Molecular and phylogenetic analysis

**Fig. 1:**
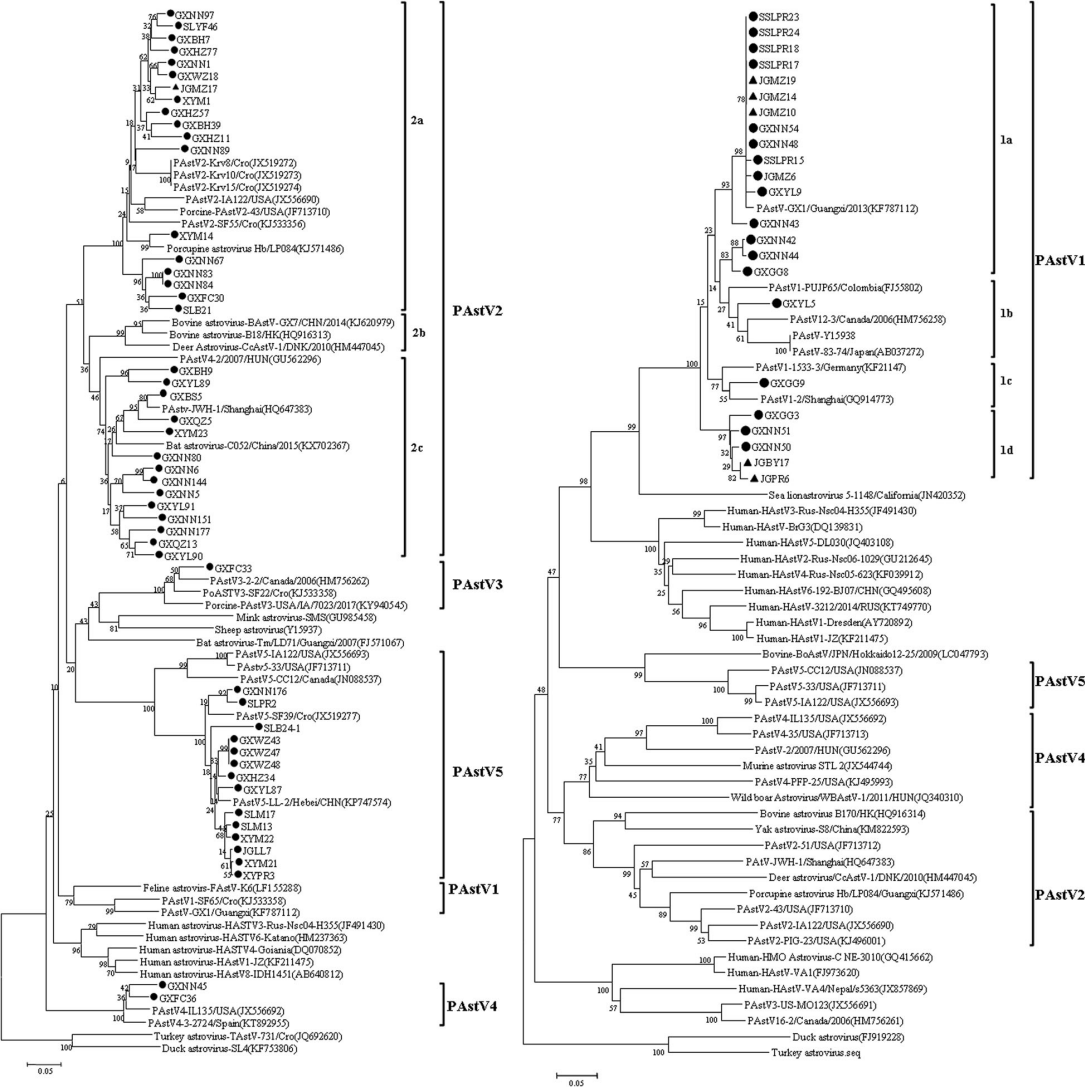
Phylogenetic trees of partial RdRp coding region (400 bp) amplified using Chu’s primers (a) and partial ORF2 coding region (183 bp) amplified using Indik’s primers (b). Two trees were generated with the neighbor-joining method using the p-distance substitution model, with 1000 bootstrap replicates and a cut-off value of 70%, with the MEGA 7.0 software. Sequences from fecal samples are marked with the dot (●)and from serum samples are marked with the triangle (▲)

Amplification and sequencing for 3ˊ-terminal conserved region of the ORF1b gene segment (400 bp) and the partial ORF2 gene (183 bp) was successful with a total of 72 sequences including 49 partial ORF1b genes of PAstV2–5 and 23 partial ORF2 genes of PAstV1. Phylogenetic trees were constructed based on these obtained nucleotide sequences compared with the selected AstV sequences from other species available in GenBank, respectively. Clearly, five clades corresponding to the five PAstV types were delineated in two phylogenetic trees (Fig. 1a and b), including PAstV1 (23/72, 31.9%), PAstV2 (32/72, 44.4%), PAstV3 (1/72, 1.4%), PAstV4 (2/72, 2.8%) and PAstV5 (14/72, 19.4%). Among these sequences, 7 serum-derived PAstV sequences were clustered into PAstV1 (6/7, 85.7%) and PAstV2 (1/7, 14.3%) from sows.

Phylogenetic analysis based on the nucleotide sequences from the 3ˊ-terminal conserved region of partial ORF1b gene segments revealed that there were four known genetic lineages circulating in the pig population of Guangxi province (Fig. 1a). In the clade of PAstV2, they were classified into three subclades (PAstV2a, PAstV2b and PAstV2c). A total of 32 strains were distant with the deer or bovine astrovirus strains (PAstV2b), but genetically closer to characterized PAstV2 strains IA122 and 43 from the USA [8] and SF55 from Croatia [5], sharing 74.9–79.6% nucleotide identity with each other. Besides, this branch included the serum-derived JGMZ17 strain, which was slightly distant from the serum-derived strain of Croatia [24] with an average nt identity of 85.9%. Another subclade (PAstV2c) clustered with strain JHW-1 from Shanghai [27] and 4–2 from Hungary [28] sharing 87.3 and 79.8% of the mean nt identities, respectively. The subclades of PAstV2a and PAstV2c were more closely related to porcupine astrovirus (KJ571486) and bat astrovirus (KX702367) detected in China respectively [29, 30], sharing 88.7 and 60.4% of the mean nt identities, respectively.

In the clade of PAstV5, 14 strains in this study shared higher identities with LL-2 from Hebei of China and SF39 from Croatia with mean nt identity of 92.6%, but with lower identities (on average 77.0% nt) with strains IA122 and 33 from the USA [8] and CC12 from Canada [31]. In addition, the strain, GXFC33, was grouped with PAstV3–2-2 from Canada, sharing 84.3% nt identity. The remaining strains GXFC36 and GXNN45 were divided into the lineage of PAstV4, sharing higher identity with stain 3–2724 from Spain with mean nt identity of 89.1%.

The 23 sequences of partial ORF2 genes were grouped into the lineage of PAstV1 (Fig. 1b). Based on its phylogenetic analysis, PAstV1 could be clustered into four subclades (PAstV1a, PAstV1b, PAstV1c and PAstV1d). In the branch of PAstV1a, six serum-derived strains clustered with other PAstV1 strains shared higher identity (on average 98.7%/96.4% nt/aa) with the strain, GX1, obtained in 2013, which was the first to describe the presence of PAstV1 viruses in blood. Four strains GXNN42, GXNN44, GXGG8 and GXYL5 were clustered with strain PUJP65 from Colombia, 12–3 from Canada, Y15938 and 83–74 from Japan, sharing 89.2%/72.1% of mean nt/aa identities. One of 23 strains (GXGG9) in the branch of PAstV1c showed higher genetic relationship with strain 1533–3 from Germany and strain 2 from Shanghai, China, sharing 90.8 and 93.5% nt identities, respectively. Additionally, five strains including two serum-derived strains (GXBY17 and GXPR6) form a single subclade (PAstV1d), sharing 88.5–96.7% nt identities with each other. Moreover, the lineage of PAstV1 showed a close relationship to other astroviruses species recovered from sea lions and humans.

### Genome features and phylogenetic analysis of the 3ˊ-end from four PAstVs

**Fig. 2:**
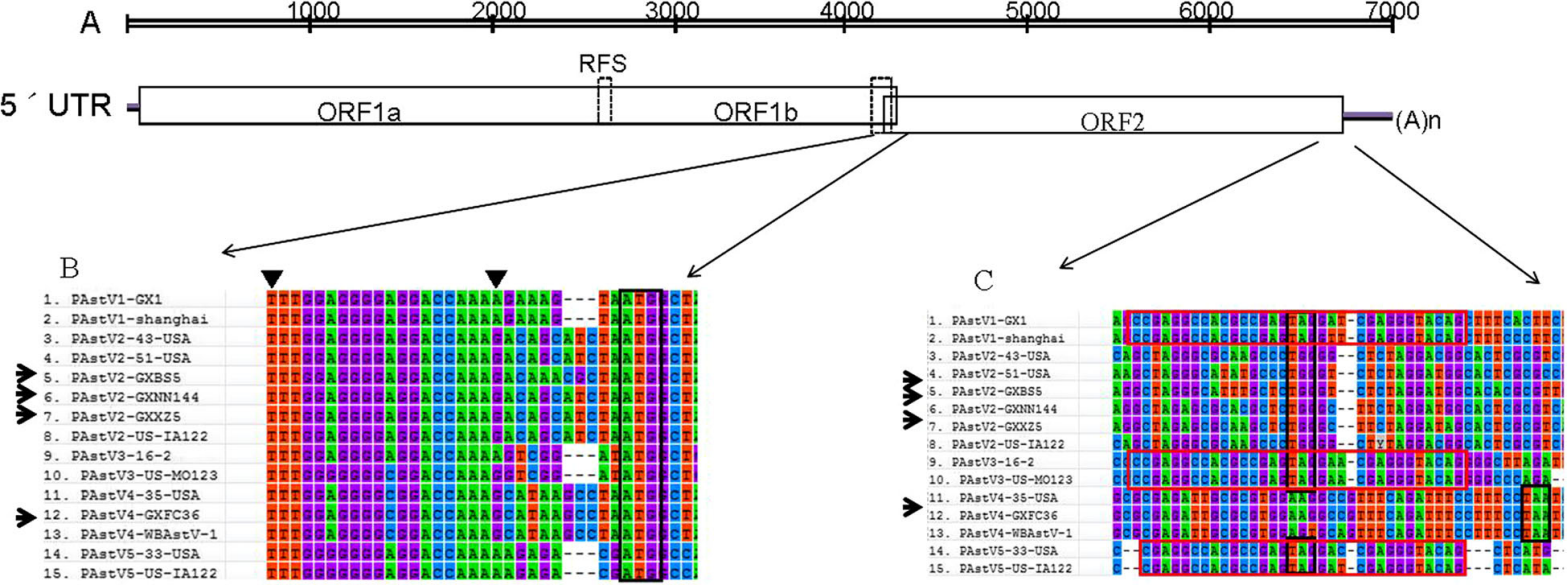
Nucleotide alignments between porcine astroviruses of this study and reference strains. (**a**) Schematic representation of the complete astrovirus. ORF1a, ORF1b and ORF2, 5ˊ UTR, 3ˊ UTR and poly A are shown. (**b**) Nucleotide alignment of the conserved sequence at the junction of ORF1b/ORF2, the proposed beginning and end of the conserved sequences was indicated by ▼; The ATG initiation codons are indicated by the black box. (**c**) Nucleotide alignment of a highly conserved motif located in the 3ˊ end of the genome of PAstVs. The conserved sequences in s2 m are indicated by the red box, and the ORF2 stop codons are indicated by black box. The arrows indicate AstV strains characterized in this study

In these PAstVs-positive samples, we were only able to successfully amplify the 3ˊ-end of the genome (about 3 kb in size) from 4 strains (GXBS5, GXXZ5, GXNN144 and GXFC36) with 3ˊ-RACE-PCR. These contain the 3ˊ-end of the ORF1b gene, the complete capsid gene (ORF2) and the 3ˊ-end untranslated regions (UTRs). The lengths of GXBS5, GXXZ5, GXNN144 and GXFC36 were 2310 bp, 2364 bp, 2322 bp and 2475 bp, respectively. Sequence alignment showed that, there is a highly conserved region (UUUGGAGGGG (A/C) GGACCAAAN8/11AUGGC (*N* = A/T/C/G)) located at the junction of ORF1b and ORF2 (Fig. 2b), which was considered to be a subgenomic promoter for RNA transcription. An insertion of 3 nt ahead of the start codon AUG resulting at N11 was different between the four strains (i.e. CGC in PAstV2-GXBS5, ATC in PAstV2-GXNN144 and PAstV2-GXXZ5, GCC in PAstV4-GXFC36). There is no a highly conserved stem-loop-II-like motif (s2 m) in the 3′-end of the genomic RNA for these four strain (Fig. 2c), which was consistent with the other PAstV2 strains and the PAstV4 strains [25, 27, 28, 32].

**Fig. 3:**
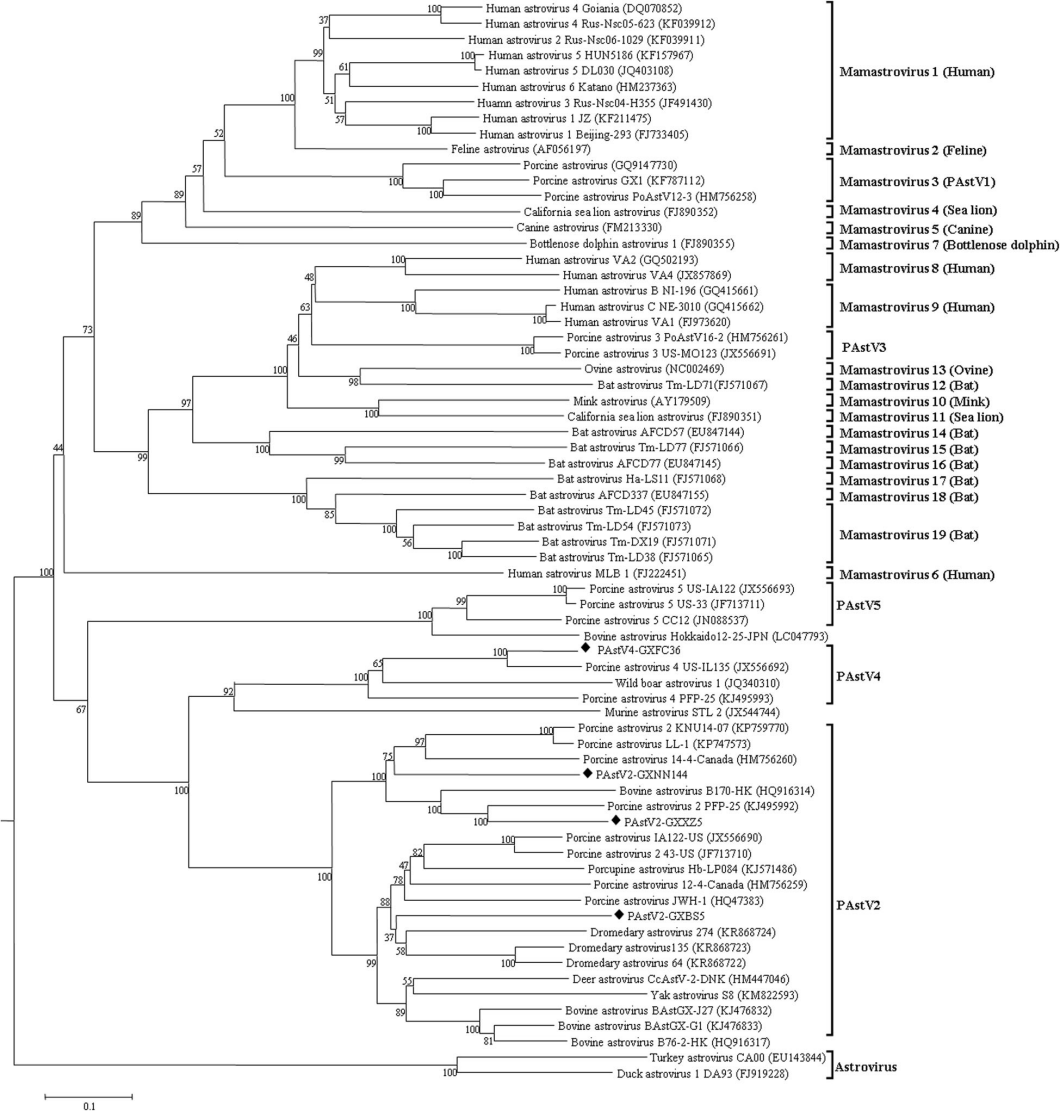
Phylogenetic tree based on the complete nucleotide sequences of the ORF2 genes (capsid) of four PAstVs. It is generated with the neighbor-joining method using the p-distance substitution model, with 1000 bootstrap replicates and a cut-off value of 70%, with the MEGA 7.0 software. The sequences of this study are marked with the rhombus (◆)

Using the comparison of 19 species in the genus *Mamastrovirus*, a phylogenetic tree based on the complete amino acid sequences of capsids was built and analyzed. It was found that four strains (GXBS5, GXXZ5, NN144 and GXFC36) in the study were classified as two distinct branches (Fig. 3), including PAstV2 and PAstV4. The strains GXBS5, GXXZ5 and NN144 were clustered into PAstV2, sharing 58.2–65.2% identity at the nucleotide level, which revealed that they were genetically close to porcupine AstV, dromedary AstV, bovine AstV from Hong Kong and other classical PAstV2 strains. In addition, the strain GXFC36 was clustered into PAstV4, compared to strain IL135 from the USA, sharing identities of 83.4 and 84.6% at the nucleotide and amino acid levels, respectively.

## Discussion

Previous reports revealed that PAstVs were highly prevalent in pigs in different countries, such as Spain (100%) [9], Austria (100%) [9], Croatia (89%) [5], Hungary (84%) [9], Canada (79.2%) [33], Italy (67.4%) [34], USA (63.9%) [8], Hunan province of China (46.3%) [12] and Czech Republic (34.2%) [35]. On the other hand, there was a comparable lower prevalence in South Korea (19.4%) [7], Germany (20.8%) [36] and Sichuan province of China (17.5%) [6]. The present overall positive rate in our study reached 56.4% (300/532), and this encompassed 28 farms from nine regions/cities without a history of diarrhea. This rate is similar to the detection results seen in the Hunan province of China. Moreover, it was found that all five PAstV types were circulating in Guangxi province of China from 2013 to 2015. It was found that PAstV2 (47.7%) showed the highest prevalence, followed by PAstV1 (26.2%) and PAstV5 (21.5%), which became predominant among five PAstVs genotypes and was detected from suckling pigs to sows. This finding is similar to the results seen in Sichuan [6], which is different from the reports from Hunan [12], suggesting that the dominance type of PAstVs varies with the different geographical locations, such as PAstV4 in the USA [8] and South Korea [7] and PAstV2 in Croatia [5]. There was low prevalence of PAstV3 (5.3%) and PAstV4 (10.5%) which existed in nursery and suckling pigs, respectively. Notably, there is no report regarding PAstV3 in China, with its first detection being noted in this study. This phenomenon is similar to what had been reported previously in other nations [5, 33, 37]. The low positive rate of PAstV4 was significantly different from the Xiao’s report, which displayed the higher prevalence of 62.3% in all age groups [8]. The application and efficiency of the different primers used for detection might be mainly responsible for the differences observed in the two studies. The specific primers for genotyping will be necessary to investigate the presence of different genotypes of PAstVs. In addition, the pig population selected, the density of selected farms, feeding environment, maintenance condition of fecal sample will result in the biases.

In the past, *Mamastrovirus* was considered to be only found in the intestine, but in recent years, many studies suggested that it also can be detected in extra-intestinal tissues, such as the brain in humans [14, 16], cattle [19, 20] and pigs [21, 22, 38], and the kidney, lung, spleen in pigs [12]. PAstV2, PAstV4 and PAstV5 have also been found in the blood of healthy pigs [24, 25]. Moser et al. showed that infection with human astrovirus could increase the intestinal barrier permeability in a Caco-2 cell culture model system, suggesting that other astroviruses might use the same way to enter the bloodstream [39]. Our study brings new knowledge about viremia caused by PAstVs which is different from genotype reported from Croatia [24]. Six serum-derived samples belonged to PAstV1 and one sample belonged to PAstV2. It kept unknown whether PAstVs could be long-standing in sows or could have been infected from nursery pigs. More paired fecal and serum samples need be investigated to show better insights into whether there is vertical transmission, possible age-restriction and extra-intestinal pathogenesis.

In order to understand genetic diversity of the strains of PAstVs in Guangxi province, 65 sequences from fecal samples were investigated, including 48 partial ORF1b genes and 17 partial ORF2 genes. Phylogenetic analysis showed that multiple distinct genotypes of PAstVs were circulating in the pig farms, which would facilitate their genetic recombination and even probably increase the occurrence of interspecies recombination events. Luo et al. indicated that PAstV1 and PAstV3 were closely related to strains found in sheep, mink, cats and humans [33], but PAstV2 was possibly restricted to pigs. Actually, in our study, PAstV2 was closer to other animal astroviruses, such as those in porcupines, bats, cattle and deer. In particular, there was higher homology (92.8% nt identity) between porcupine astrovirus and PAstV2-XYM14, further suggesting they could share the same ancestral origin, but whether there were past cross-species transimission between porcupine and swine by yet-undentified intermediate hosts required further investigation [30].

Further 3′ partial genomic sequencing and characterization of four selected stains revealed the genetic diversity that exists. They only shared 58.2–65.2% identity at the nucleotide level between PAstV2-GXBS5, PAstV2-GXXZ5 and PAstV2-NN144. Previous studies have shown that most of the AstVs strains contain a conserved stem-loop II-like motif (s2 m) near the 3ˊ-UTR [25, 31, 40] which was thought to play an important role in viral life cycle [41]. However, our study found the lack of s2 m in PAstV2 and PAstV4 strains, in agreement with the previous reports [25, 33, 42], so whether the lack of s2 m will affect the biological function, or whether the virus could compensate by forming stable secondary structures in the different regions of their 3ˊ-UTR [33], remains to be confirmed by further studies.

## Conclusion

Our present work shows a comprehensive overview of PAstVs for the first time in Guangxi province, China. In particular, the results reveal the existence of all five known lineages in the pig population with a high prevalence of PAstV2, PAstV1 and PAstV5 in animals of different ages and newly described PAstV3 presence in China. Importantly, this study also proved that porcine astroviruses exist in blood with the dominance of PAstV1, adding to the reported genotypes (PAstV2, PAstV4 and PAstV5), which suggested there would be wider presence of different PAstV genotypes in blood, possibly resulting in a more complicate pathogenesis outside the enteric system. Furthermore, phylogenetic analysis of the partial genes and molecular characterization revealed high genetic heterogeneity and more information about 3′ partial genome. The strains of PAstV2 and PAstV3 were close to known astrovirus species from many different animals suggesting different ancestral origins or occurrence of interspecies transmission. More PAstVs whole genome sequences will be needed in order to fully understand the evolution and ecology of the *Astroviridae* family of viruses.

## Materials and methods

### Samples collection and processing

A total of 532 composite fecal samples (fecal swabs and feces) were collected from pigs of 28 different scale farms in Guigang, Yulin, Baise, Baihai, Qinzhou, Wuzhou, Hezhou and Fangchenggang of Guangxi Zhuang Autonomous Region (Guangxi province) from 2013 to 2015, including suckling pigs (*n* = 141), nursery pigs (*n* = 145), fully grown pigs (*n* = 122) and sows (*n* = 124). In addition, a total of 120 serum samples were randomly collected only from two industrialized pig farms (designated as farm A and farm B, respectively) in 2015, including 60 serum samples from each farm. Sixty serum samples were collected from each pig farm and these included suckling pigs (*n* = 20), nursery pigs (*n* = 10), fully grown pigs (*n* = 10) and sows (*n* = 20). The fecal samples were placed into 10 mL centrifuge tubes and diluted with 1 × Phosphate Buffered Saline (PBS, pH 7.4) containing 200 U/ml penicillin, 200 mg/ml streptomycin. Samples were homogenized by vortexing for 5 min at room temperature and centrifuged at 12,000 rpm for 15 min at 4 °C. Viral RNA was extracted from 20% (w/v) fecal supernatants and serum samples using the RNAiso PLUS kit (Takara Bio, Inc., Dalian, China). 300 μL of fecal homogenate were prepared for viral extraction and viral RNA was stored at − 80 °C until needed.

### Detection for PAstVs using nested RT-PCR

cDNA was generated from RNA using PrimeScript Reverse Transcriptase and random hexamers (Takara Bio, Inc., Dalian, China) from 8 μL of RNA sample according to the manufacturer’s instructions. The nested primers for PCR were used to amplify the partial RNA-dependent RNA polymerase (RdRp) gene specific for PAstV2-PAstV5 [40]. Due to the low sensitivity to PAstV1, another set of primers specific for the partial ORF2 gene were employed to detect PAstV1 [43], respectively (Table 2). To avoid the possible contamination, the fecal samples and serum were processed and detected separately and the double negative controls were set up in RT-PCR. Once we obtained the PAstV-positive samples, which will be double checked. The PCR cycling conditions using the two different sets of primers were as described previously [33]. PCR products were purified and ligated to pMD18-T vector (Takara, Japan). The plasmids were identified by double digestions with Qcut BamH I and Qcut Hind III (Takara, Japan) and sequenced as described previously [44].

**Table 2:** Primers used in this study

Primer name	Primers sequences(5′-3′)	Genes for amplification	Reference
GSP-2A	CTAAGTACGTGCTCATGCCATCT	Complete ORF2	In this study
GSP-2B	GATGACAGGCTTACAACCACTCC	Complete ORF2	In this study
GSP-4A	GTATGTTATGATGCCGAGTGG	Complete ORF2	In this study
GSP-4B	TTGACCCGTTATCCAATCTTACCAG	Complete ORF2	In this study
AST248F	GTGTCACAGGTCCAAAACCAGCAAT	5′ end of ORF2	Indik et al. [43]
AST665R	TGGTGTTCGTCAACCACCAGCC	5′ end of ORF2	Indik et al. [43]
ASTneF	CTCGAGGCATGCATCCTCAC	5′ end of ORF2	Indik et al. [43]
ASTneR	AAGAGAAGCACGGACAACTG	5′ end of ORF2	Indik et al. [43]
panAV-F11	GARTTYGATTGGRCKCGKTAYGA	Partial RdRp	Chu et al. [40]
panAV-F12	GARTTYGATTGGRCKAGGTAYGA	Partial RdRp	Chu et al. [40]
panAV-F21	CGKTAYGATGGKACKATICC	Partial RdRp	Chu et al. [40]
panAV-F22	AGGTAYGATGGKACKATICC	Partial RdRp	Chu et al. [40]
panAV-R1	GGYTTKACCCACATICCRAA	Partial RdRp	Chu et al. [40]

### Amplification of 3ˊ-end of selected PAstVs using 3ˊ-RACE PCR

The 3ˊ-end genes of selected PAstVs were amplified using a commercial 3ˊ-RACE kit (Takara, Japan). According to the manufacturer’s instructions, two groups specific forward nested primers based on the ORF1b sequence were designed (Table 2). Among these, the primer GSP-2A (Outer specific primer) and GSP-2B (Inner specific primer) were used for capsid protein region amplification of the PAstV2 strains (GXBS5, GXXZ5 and GXNN144) in this study. The primer GSP-4A (Outer specific primer) and GSP-4B (Inner specific primer) were used for the PAstV4 strain (GXFC36) in this study.

Briefly, the viral RNA was reverse-transcribed into cDNA using the M-MLV reverse transcriptase (RNase H-) and 3ˊ-RACE adaptor primer (provided in the kit). Then the 3ˊ-end was amplified using nested PCR. The first PCR reaction was performed using the outer specific primer and the 3ˊ-RACE outer primer (complementary to the outer part of 3ˊ-RACE adaptor primer) provided in the kit and the reaction was pre-degenerated at 94 °C for 5 min, followed by 20 cycles at 94 °C for 30s, 58 °C for 30s, 72 °C for 2 min 30s and final extension at 72 °C for 10 min. The first-round PCR product was used as the template for the second-round PCR reaction which was performed using an inner specific primer and a 3ˊ-RACE inner primer (complementary to the inner part of 3ˊ-RACE adaptor primer) provided in the kit. The reaction conditions were the same as the first round PCR except the cycles were increased to 35 cycles. PCR products were purified, cloned and sequenced as described previously [44].

### Sequence alignment and phylogenetic analysis

The first comparisons of PAstV sequences obtained with AstVs reference strains were performed by BLAST program in NCBI (http://blast.ncbi.nlm.nih.gov/Blast.cgi). At the same time, the nucleotide sequences obtained in this study were aligned with published AstVs reference strains by the ClustalW (1.6) method using MEGA 7.0 software. The same software was used to reconstruct phylogenetic trees from evolutionary distances using the Neighbor-Joining (NJ) method with p-distances for nucleotide sequences. The clustering stability of the NJ tree was evaluated by the bootstrap test value of 1000 replicates. Astrovirus sequences characterized in this study were deposited to GenBank under accession numbers KM211520 to KM211529, KY230626 to KY230653 and KY412128 to KY412139 and MH064173 to MH064176 for partial RNA dependent RNA polymerase, KY412101 to KY412123 for partial capsid protein sequences and KY412124 to KY412127 for the complete capsid protein sequences.

## Data Availability

All the nucleotide sequences in this study are available in GenBank under following accession numbers: KM211520 to KM211529, KY230626 to KY230653 and KY412128 to KY412139 and MH064173 to MH064176 for partial RNA dependent RNA polymerase, KY412101 to KY412123 for partial capsid protein sequences and KY412124 to KY412127 for the complete capsid protein sequences.

## Abbreviations

3ˊ-RACE: 3ˊ rapid amplification of cDNA ends
AstVs: Astroviruses
PAstVs: Porcine astroviruses
RT-PCR: Reverse transcriptase-polymerase chain reaction
UTR: Untranslated regions
